# Structural, Thermodynamic and Enzymatic Characterization of *N*,*N*-Diacetylchitobiose Deacetylase from *Pyrococcus chitonophagus*

**DOI:** 10.3390/ijms232415736

**Published:** 2022-12-12

**Authors:** Katarzyna Biniek-Antosiak, Magdalena Bejger, Joanna Śliwiak, Daniel Baranowski, Ahmed S. A. Mohammed, Dmitri I. Svergun, Wojciech Rypniewski

**Affiliations:** 1 Institute of Bioorganic Chemistry, Polish Academy of Sciences, Noskowskiego 12-14, 61-704 Poznań, Poland; 2 European Molecular Biology Laboratory (EMBL), Hamburg Unit, DESY, Notkestrasse 85, D-22607 Hamburg, Germany

**Keywords:** *N*,*N*-diacetylchitobiose deacetylase, chitin, *Pyrococcus chitonophagus*, hyperthermophile, crystallography, protein structure, thermodynamics, SAXS, DSC, ITC, NMR

## Abstract

Chitin is a major source of energy and macroelements for many organisms. An important step in its degradation is the deacetylation of chitin or its fragments. Deacetylase from the extremophile *Pyrococcus chitonophagus* has been analyzed by X-ray crystallography, small-angle X-ray scattering, differential scanning calorimetry, isothermal titration calorimetry and NMR to determine its structure, thermodynamics and enzymatic properties. It is a hexameric, zinc-containing metalloenzyme that retains its structural integrity up to temperatures slightly exceeding 100 °C. It removes the acetyl group specifically from the non-reducing end of the sugar substrate. Its main substrate is *N*,*N*-diacetylchitobiose but it also active, at a reduced level, toward *N*-acetyl-d-glucosamine or a trimer of *N*-acetyl-d-glucosamine units. Crystallographic analysis includes the structure of the enzyme with its main substrate approaching the active site in a monodentate manner, replacing the single water molecule that is bound at the Zn^2+^ cation when the ligand is absent. The Zn^2+^ cation remains tetrahedrally coordinated, with three of its ligands provided by the protein’s conserved His-Asp-His triad. The crystal structures are consistent with the reaction mechanism proceeding via an anhydride intermediate. Hydrolysis as the first step cannot be ruled out in a hydrated environment but no defined ‘hydrolytic water’ site can be identified in the analyzed structures.

## 1. Introduction

After cellulose, chitin is the second most abundant biopolymer on Earth. It consists of *N*-acetyl-d-glucosamine (GlcNAc) residues linked by β-(1→4) covalent bonds. It is the primary component of cell wall in fungi, and exoskeletons in crustaceans and insects. It is also synthesized and utilized by mollusks, some fish and amphibians. The total chitin production in the aquatic biosphere is estimated at 10^9^ tons per year [1]. Compared to cellulose, chitin is much underutilized by humans, even though it is more valuable as biomass because it contains nitrogen in addition to carbon. Chitin is utilized primarily by microorganisms, which recycle most of the organic detritus, known as ‘ocean snow’, before it sediments on the see floor. They use specialized enzymes, such as chitinases for cleaving the β-(1→4) glycosidic bonds, and deacetylases, which turn the *N*-acetyl-d-glucosamine residues into glucosamine.

*Pyrococcus chitonophagus*, previously known as *Thermococcus chitonophagus*, is a hyperthermophilic anaerobic archaeon isolated from a hydrothermal vent off the Mexican west coast, at a depth of 2600 m. It was shown to grow chemoorganoheterotrophically, i.e., ingesting organic compounds as a source of energy. It uses chitin as a sole carbon source, indicating an adaptation for recycling this polysaccharide within its specific microenvironment [2]. *P. chitonophagus* genome contains genes for a set of chitinolytic enzymes including three chitinases, a diacetylchitobiose diacetylase (Dac) and *exo*-β-d-glucosaminidase (GlmA) [3].

Different chitin deacetylases act either on chitin, to turn it into chitosan, the de-*N*-acetylated product, (EC 3.5.1.41) [4], or they act on shorter GlcNAc oligomers, most commonly (GlcNAc)_2_, the main product of chitin degradation by a chitinase. Many deacetylases are grouped into either the carbohydrate esterase (CE) family 4 (CE-4), when they act on the reducing end of the carbohydrate oligomer (EC 3.5.1.105) or CE-14 when they act on the nonreducing end (EC 3.5.1.136).

Recent results of research on CE-14 deacetylases include crystal structures of the enzymes from *Pyrococcus horikoshii* and *Pyrococcus furiosus* [5]. The proteins have been described in complexes with an acetate ion, i.e., the product of the enzymatic reaction, with a reaction intermediate analog [6], with phosphate bound near the active site, and with Cd^2+^ ions substituting Zn^2+^ [7]. Here, we present an extended structural, thermodynamic and enzymatic study of a related archaeal deacetylase *Pyrococcus chitonophagus*. The results include the structure of the enzyme with its substrate.

## 2. Results and Discussion

### 2.1. Oligomeric State and the Overall Structure

**Figure 1:**
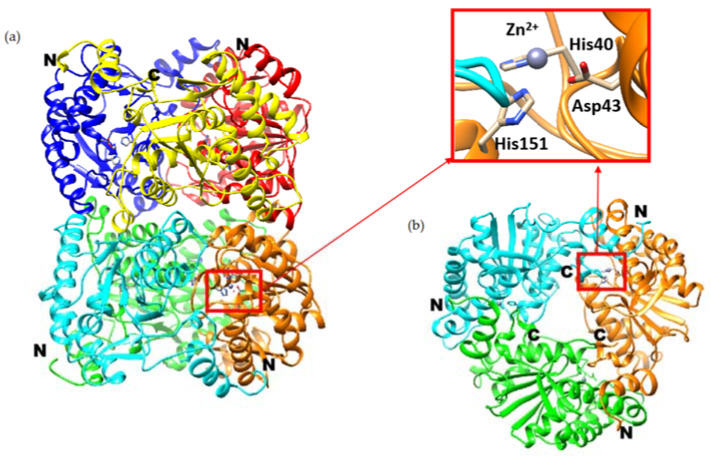
Ribbon diagram of a *Pch*-Dac hexamer, i.e., two trimers stacked back-to-back (**a**). A view along the long molecular axis, showing the channel in the middle (**b**) (only the three foremost polypeptide chains are shown). The Zn^2+^ cations (blue spheres) and their chelating protein residues are drawn in detail, indicating the active sites. The N-and C-termini are marked. The inset shows a close-up view of the metal-binding triad.

*Pch*-Dac crystallizes as ‘a dimer of trimers’, with two doughnut-shaped trimers stacked back-to-back along a molecular three-fold axis (Figure 1a), with a 8–10 Å channel through the middle of this assembly (Figure 1b). Three crystal forms were analyzed, including monoclinic crystals of the protein-substrate complex (*Pch*-Dac-lig), having two protein hexamers in the crystal’s asymmetric unit and trigonal crystals of the unliganded protein (*Pch*-Dac), having half a hexamer in the asymmetric unit, with the other half being related by the crystallographic two-fold axis. Orthorhombic crystals, *Pch*-Dac-anom, used for identifying the metal cation, have one hexamer in the asymmetric unit. The same hexameric assembly is observed regardless of crystal packing and probably represents the biologically relevant oligomeric form of the enzyme.

The oligomerization of the protein was also investigated in solution using small-angle X-ray scattering (SAXS) and the study showed that the protein in solution was predominantly hexameric, with a minor component attributed to a higher state of oligomerization. The volume fractions of the two components in the dilution series, and the corresponding goodness-of-fit (χ^2^) are presented in Table S1 and the typical fit (to the highest concentration data) is displayed in Figure S1. Fitting the scattering curve, using the hexameric model supplied by crystallography, accounted for 84–87% of the scattering, while the remaining signal was attributed to a hypothetical ‘dodecameric’ form. Two neighboring *Pch*-Dac hexamers had been extracted from the trigonal crystal lattice and used to improve the curve fitting. The improvement indicated that a fraction of the molecules in solution associated to form larger assemblies. Surprisingly, the estimated amount of aggregation was independent of protein concentration. This is difficult to explain owing to thermodynamic considerations. It is possible that the protein preparation underwent partial deterioration during transport and storage on ice. Nevertheless, the combination of SAXS and analytical on-line size-exclusion chromatography (SEC) successfully isolated the hexamer signal. The collected signal from the main peak in the chromatogram (Figure S2) was fitted well by the scattering computed from the crystallographic hexamers, with χ^2^ = 1.2 (see also Table S1). The small pre-peak in the chromatogram (Figure S2) does not contain the signal from dodecamers but rather shows some larger aggregates. This confirms that hexamers represent the most stable fraction in solution and probably form the biologically relevant species.

**Figure 2:**
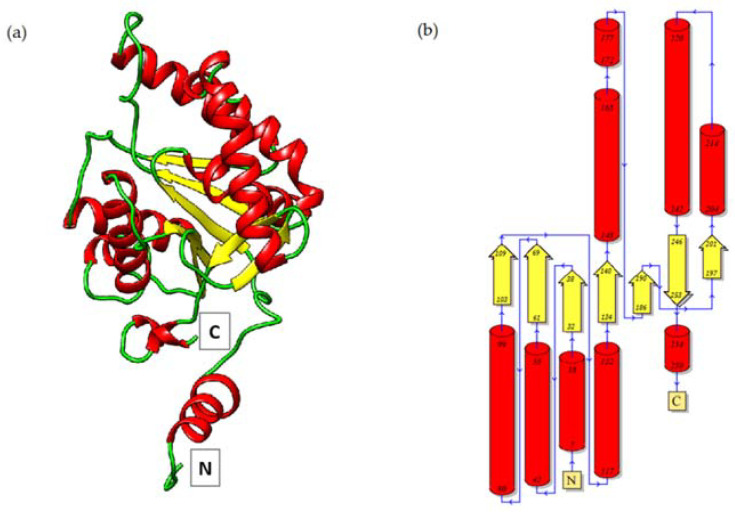
Ribbon diagram (**a**) and topology of the secondary structure (**b**) of a *Pch*-Dac subunit. The N- and C-termini are indicated.

Each *Pch*-Dac subunit is an α/β domain comprising seven β-strands (of which one is anti-parallel to the others) sandwiched between α-helices (Figure 2). Approximately 20 N-terminal residues and 13 C-terminal residues form an extension reaching to the neighboring subunit and forming part of its substrate-binding site. Each *Pch*-Dac subunit contains a metal cation bound by a conserved His-Asp-His metal-ion-binding triad (His40-Asp43-His151, Figure 1 inset), characteristic of the Carbohydrate Esterase Family 14 (CE-14), of which the most similar known structures are diacetylchitobiose deacetylases from *Pyrococcus horikoshii* and *Pyrococcus furiosus* [5].

### 2.2. Thermal Stability and Melting Profile of Pch-Dac

**Figure 3:**
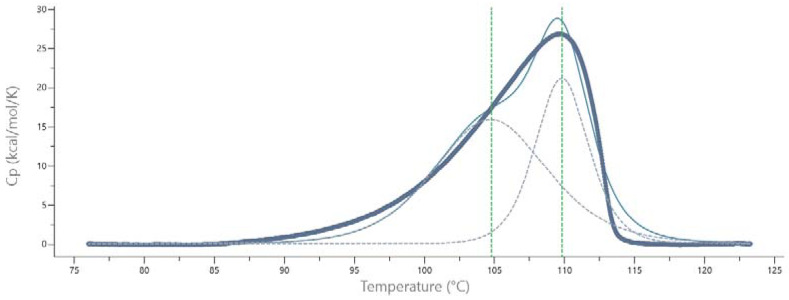
DSC melting curve of *Pch*-Dac (1.24 mg/mL) at the scan rate of 60 °C/h, in buffer B: 25 mM HEPES pH 7.5, 200 mM NaCl, 10 μM ZnCl_2_ (thick solid line). A possible interpretation of the asymmetric shape of curve is shown (dashed lines) as two thermal events with maxima at 105 and 110 °C. A summation of the two peaks is shown as thin solid line.

The calorimetric (DSC) spectrum of *Pch*-Dac contains a distinct single peak with a maximum at 110 °C, indicating the melting temperature of the protein (Figure 3). The peak is asymmetric, suggesting that unfolding of the protein is not a simple one-step process. The shoulder of the peak could be interpreted as a minor thermal event taking place at the temperature 105 °C, possibly corresponding to deoligomerization prior to unfolding of the polypeptide chains [8].

Reversibility tests, consisting of cooling the unfolded protein, showed that denaturing of *Pch*-Dac was irreversible under the experimental conditions (1.8 mg/mL, Buffer A: 20 mM TRIS pH 7.4, 200 mM NaCl, scan rate 60 °C/h in both directions, in the range 40–130 °C).

### 2.3. Identifying the Prosthetic Group by Microcalorimetry and Anomalous X-ray Scattering

Each subunit contains a metal cation which takes part in substrate binding and catalysis. By analogy to related structures the cation is expected to be Zn^2+^ [5]. However, Cd^2+^ was also found to bind in a related protein structure [7].

**Figure 4:**
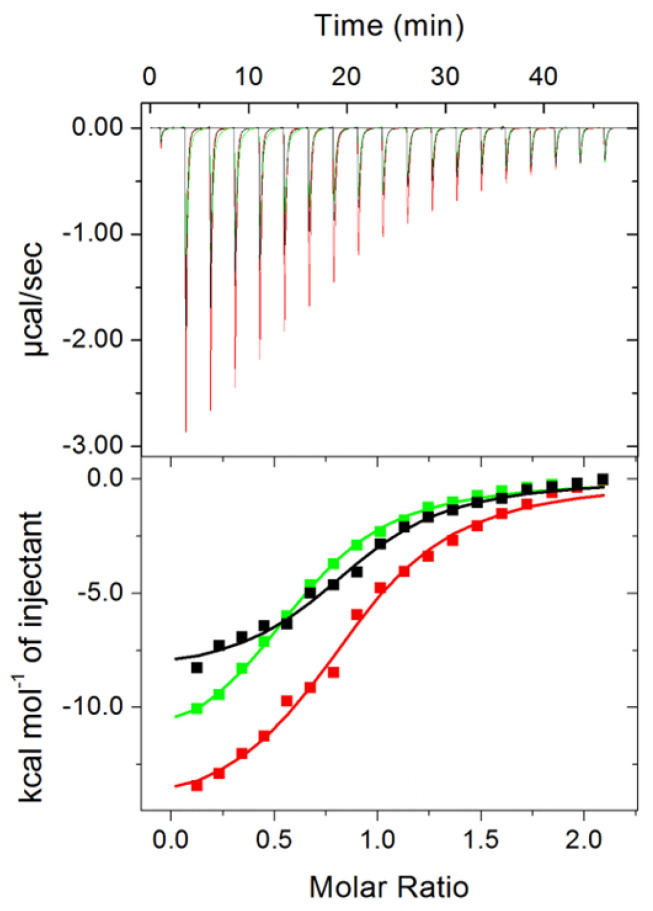
Representative raw data (upper panel) and integration of the peaks with the best fit of ‘one set of sites’ model (bottom panel), obtained after titrations of *Pch*-Dac (~110 µM) with 1.2 mM Zn^2+^ (black), Cd^2+^ (red) or Ni^2+^(green).

The binding affinity of *Pch*-Dac for Zn^2+^, Cd^2+^ and Ni^2+^ was measured by microcalorimetry. Thermodynamic parameters were calculated from the titration curves. The study showed that the K_d_ values of Zn^2+^, Cd^2+^ and Ni^2+^ binding were similar, within the estimated measurement errors (9.5 ± 1.3, 11.0 ± 1.3 and 12.7 ± 1.0 µM, respectively) (Table 1, Figure 4). The enthalpy contribution in the binding event is largest in the case of Cd^2+^ and lowest for Zn^2+^. The stoichiometry Cd^2+^ and Zn^2+^ could be approximated to 1. The lower stoichiometry (0.6) of Ni^2+^ binding could be the result of sub-optimal protein conformation (which deteriorates over time) or by the fact that the removal of cations by *N*,*N*,*N*′,*N*′-tetrakis(2-pyridinylmethyl)-1,2-ethanediamine (TPEN) was incomplete and Ni^2+^, which has a lower affinity for the protein, did not occupy every metal binding site during the titration. Nevertheless, these experiments indicate that Ni^2+^, or possibly other divalent cations, in high molar excess are able to displace Zn^2+^.

The presence of Zn^2+^ in the crystal structure of *Pch*-Dac was confirmed by X-ray anomalous dispersion. Energy scan performed on the *Pch*-Dac-anom crystals revealed X-ray absorption profile characteristic of zinc, while anomalous density maps clearly identified the Zn^2+^ sites as peaks at the level of 18–25 r.m.s.d.

### 2.4. Substrate Binding

**Figure 5:**
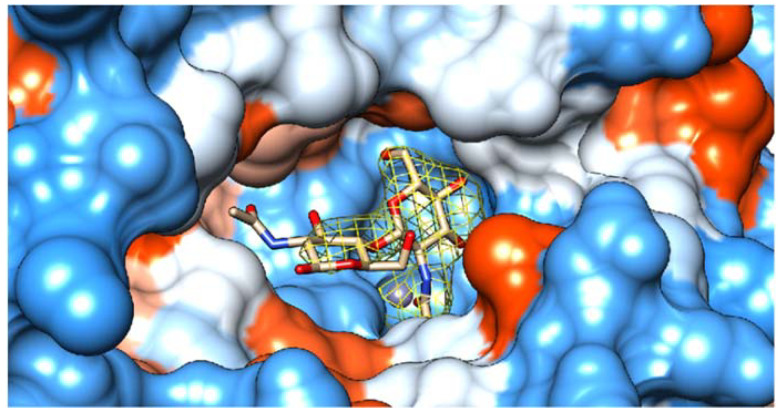
Surface view of the substrate-binding pocket of *Pch*-Dac with bound (GlcNAc)_2_. The color of the protein surface indicates the electrostatic charge: negative (red), positive (blue) and neutral (white). The Zn^2+^ ion is shown as a blue sphere. The bound substrate is drawn as sticks. The contours show the 2F_o_-F_c_ electron density of the ligand, at 1σ level.

A (GlcNAc)_2_ substrate molecule was found in each substrate-binding cavity of the 12 crystallographically independent subunits in the *Pch*-Dac-lig structure. The electron density clearly indicated two connected sugar rings (Figure 5 and Figure S3).

**Figure 6:**
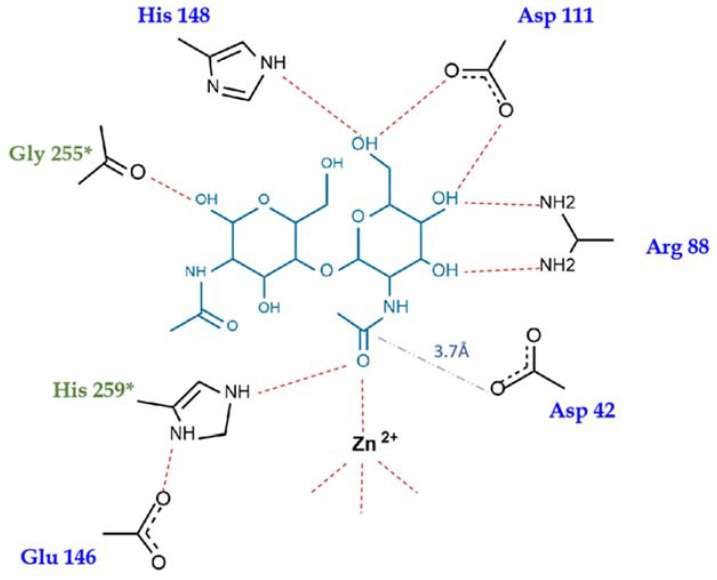
Scheme of (GlcNAc)_2_ substrate binding to *Pch*-Dac. Hydrogen bonds are indicated by red dotted lines, the distance from Asp42 to the C atom at the scissile bond is marked by a blue dashed line. Residues that form part of the substrate binding site, that come from the neighboring subunit, are marked with *.

The bound carbohydrate is oriented with its non-reducing end toward the active site, consistently with other enzymes of the CE-14 family and differently from CE4 enzymes which act on the reducing end of their sugar substrates. The GlcNAc moiety that is closer to the active site is held in place by a network of hydrogen bonds to the surrounding protein residues (Figure 6). The *N*-acetyl group is directed toward Zn^2+^ and its carbonyl oxygen atom completes the tetrahedral coordination sphere of the Zn^2+^ ion (the Zn^2+^ to O distance is 2.1 ± 0.03 Å). The substrate’s *N*-acetyl group is flanked by Asp42 and His259* (* denotes that the residue comes from the neighboring subunit). Its position is equidistant from the two flanking residues (3.5 ± 0.2 Å from the carbonyl oxygen to Nε2 of His259 and to Oδ1 of Asp42). Interestingly, Asp42 is consistently the most outlying residue on the Ramachandran plot (Figure S4). It lies at the beginning of a short helix preceded by a Pro residue. The methyl part of the *N*-acetyl group of the substrate is lodged in a hydrophobic pocket formed by Ile46, Phe219, Trp227 and Ile260*.

The position of the other sugar moiety is less well defined, but can be fitted in the electron density. It makes one H-bonding interaction with the protein: between the O1 hydroxyl group and the carbonyl oxygen atom of Gly255 of the neighboring subunit.

It is unusual in crystallography to find a substrate molecule in an enzyme’s active site. One would rather expect the substrate to be processed by the enzyme before the diffraction measurements can be carried out. Possible factors that could explain the observed stability of the substrate-enzyme complex are: (1) sub-optimal temperature (20 °C) and pH (6.0) of the solution containing the crystal. The optimal temperature for the reaction is above 75 °C, and optimal pH is 7.5 (see ITC results below). (2) A large excess of fresh substrate present in the cryosolution in which the crystal was immersed just before being frozen. (3) The diffraction measurements were performed in cryogenic conditions (100 K).

**Figure 7:**
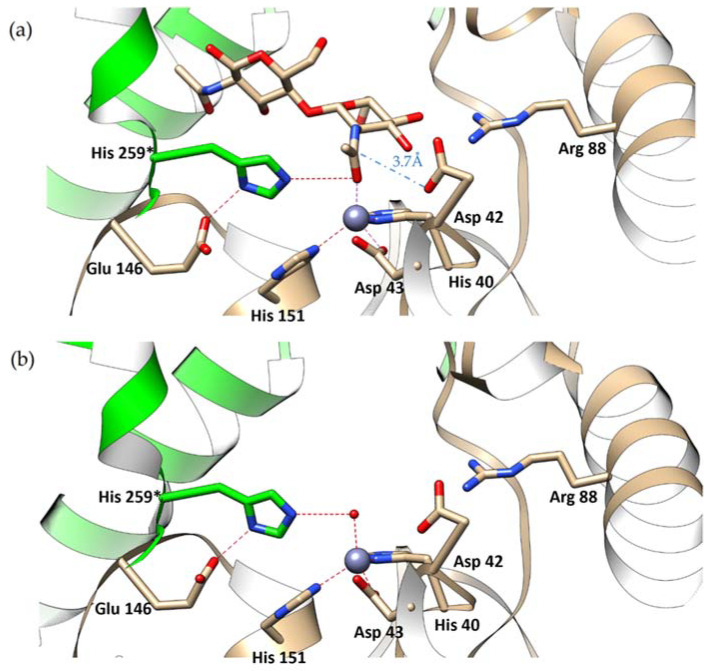
Details of (**a**) the substrate-binding site of *Pch*-Dac-lig structure with bound (GlcNAc)_2_ and (**b**) *Pch*-Dac structure with a single water molecule bound to Zn^2+^. The Zn^2+^ cation is drawn as a blue sphere; water is a small red sphere. Hydrogen bonds are marked with red dotted lines. The distance from Asp42 to the C atom of the scissile bond of the substrate is indicated by a blue dashed line. Asterisk (*) marks His259 that forms part of the substrate-binding site although it comes from a neighboring protein subunit. A comparison of the substrate-bound and unliganded structures shows that the presence of the substrate does not seem to make any significant change to the structure of the protein.

In *Pch*-Dac and *Pch*-Dac-anom, there was no ligand in the substrate-binding sites and the coordination sphere of Zn^2+^ consists of the three conserved His-Asp-His residues and a single water molecule that occupies the same position as the carbonyl oxygen of the substrate’s *N*-acetyl group in the *Pch*-Dac-lig complex. Thus, with or without the substrate, the Zn^2+^ cation is coordinated tetrahedrally (Figure 7).

This calls for some discussion with regard to the reaction mechanism. Two different models have been proposed in the literature concerning the broad family of hydrolytic enzymes containing Zn^2+^, that include proteinases/peptidases and deacetylases (see [9] and references therein). In one model, the reaction involves a hydrolytic water molecule as the first step, leading to a tetrahedral intermediate product. An alternative model involves a nucleophilic attack by a conserved Glu or Asp, with the formation of an anhydride intermediate, followed by hydrolysis as the second step. Different studies, by various techniques (NMR, crystallography, QM) of the related enzymes support one model or the other [9].

Related crystal structures described in the literature show the following:

1. The unliganded lipoglycopeptide antibiotic deacetylase (Orf2*) has two clear water molecules bound to the Zn^2+^ cation, thus making the Zn^2+^ pentacoordinated (PDB ID 3DFF) [10]. This is consistent with the model according to which one water molecules is displaced by the substrate while the second is the ‘hydrolytic water’ acting as the nucleophile. This enzyme, however, has a different architecture of the binding site from *Pch*-Dac. The gap between the Asp and His residues, flanking the active site, is wider in Orf2* by approximately 2 Å (ca 9 Å), compared to *Pch*-Dac (7 Å), probably due to the different nature of its substrate. The wider substrate binding site in Orf2* can more easily accommodate the two water molecules. An amino acid sequence comparison of the two enzymes shows that whereas the Asp residues come from corresponding places, the His residues are unrelated. The monomeric Orf2* is self-contained, whereas in the oligomeric *Pch*-Dac the catalytic His259* comes from the neighboring subunit.
2. The two deacetylases from P*yrococcus horikoshii* and *Pyrococcus furiosus* that are closely related to *Pch*-Dac have been described with Zn^2+^ or Cd^2+^ in the metal-binding site and different ligands [5,6,7]. In the complex with the reaction intermediate analog (MPG), two oxygen atoms of the ligand approach the active site but they interact asymmetrically: one makes a direct interaction with Zn^2+^ (the average distance is 1.9 Å), while the other is more distant (2.8 Å). In these complexes, Zn^2+^ appears tetrahedrally coordinated [6]. Asymmetric interactions with Zn^2+^ are also observed in complexes with the phosphate: one of the oxygen atoms is closer to the cation (1.9 Å) than the other (3.1 Å) (PDB ID 3WL3) and in a complex with an acetate ion (1.9 and 2.4 Å) (PDB ID 3WE7) [5]. In the absence of ligands, the six crystallographically independent subunits contain water molecules that can be interpreted as either partially disordered or they can be modeled as a single site near Zn^2+^ or as two water molecules but also at different distances to the Zn^2+^ cation (PDB ID 4XM0, 4XM2) [7]. Overall, Zn^2+^ shows a clear tendency to be tetrahedrally coordinated, taking one ligand in addition to the conserved His-Asp-His triad. This is different from the Cd^2+^-substituted proteins, in which the Cd^2+^ cations show a tendency to be octahedrally coordinated, with some distortions to this geometry or one ligand missing in the geometrically octahedral six-ligand coordination shell (PDB ID 3Wl4, 4XM1, 4XLZ) [5,7].

In summary, the Zn^2+^ cations in *Pch*-Dac and in closely related enzymes show a propensity to be tetracoordinated and external ligands approach it in a monodentate rather than bidentate manner. This disfavors the model, according to which the enzyme in its resting state has two water molecules at the Zn^2+^ ion, one of which is displaces by the substrate’s carbonyl oxygen, while the other acts as the hydrolytic water. In *Pch*-Dac, the Zn^2+^ is clearly tetracoordinated and the single water molecule is displaced by the substrate. This does not exclude the possibility of hydrolysis as the first step of the reaction, as the environment is of course fully hydrated. There is, however, no defined site for a water molecule, from which it could carry out the hydrolytic attack, and it is not clear how this water could approach the substrate that is lodged equidistantly between Asp42 and His259* (ca. 3.5 Å from each). It seems necessary for the structure to change before the hydrolysis could take place. One could also consider the possibility of the reaction proceeding through an anhydride intermediate.

### 2.5. Enzyme Kinetics

The activity of *Pch*-Dac was characterized by microcalorimetry (ITC) against three substrates: GlcNAc, (GlcNAc)_2_ and (GlcNAc)_3_. Table 2 summarizes the results obtained at pH 6.5, at 75 °C. They clearly indicate that the enzyme acts on (GlcNAc)_2_ with the highest efficiency.

Although pH 6.5 seems not optimal for the activity against (GlcNAc)_2_, it was the optimal common pH for the comparative study of the different substrates. With GlcNAc at pH 7.5 and 8.5, the determination of kinetic parameters was difficult due to a complicated mechanism of the reaction, i.e., at lower concentrations of the substrate (up to ~15 mM) the reaction was endothermic and in the subsequent titration steps it was exothermic. At pH 6.5 the reaction was exothermic (interestingly, with (GlcNAc)_2_ and (GlcNAc)_3_ the reactions were endothermic). We tested the activity on GlcNAc also at lower pH (5.5) and it resulted in similar kinetic parameters (K_M_ = 17.7 ± 1.9 mM, k_cat_ = 160 ± 6 s^−1^).

Table 3 compares the kinetic parameters of (GlcNAc)_2_ deacetylation under various pH conditions. It shows that pH in the case of (GlcNAc)_2_ has no dramatic effect on the enzyme’s activity. The enzyme is most efficient at pH 7.5, nearly as efficient at pH 6.5 and half as efficient at higher pH 8.5.

**Figure 8:**
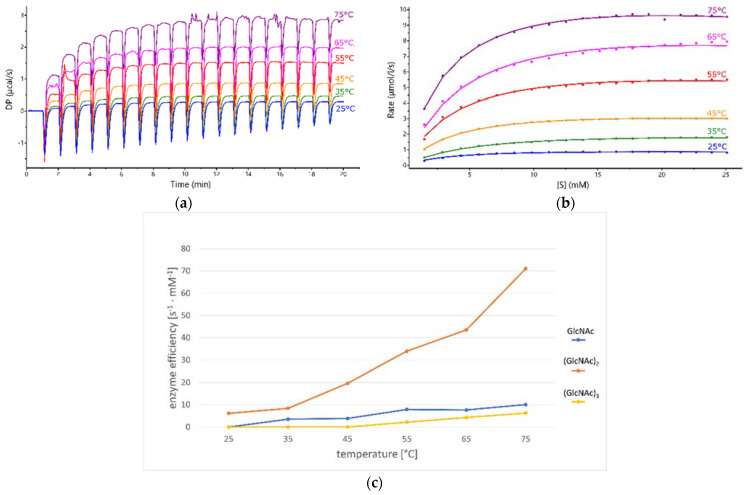
(**a**) The raw kinetic data obtained by ITC multiple injection method of measuring the kinetics of (GlcNAc)_2_ deacetylation at different temperatures. (**b**) The heat-flow levels after each injection of 2µL of 150 mM substrate, transformed into reaction rates and plotted against (GlcNAc)_2_ concentration; Michaelis-Menten equation was fitted to the obtained curves. (**c**) Graph of the dependence of enzyme efficiency on temperature for GlcNAc, (GlcNAc)_2_ and (GlcNAc)_3_ substrates.

We followed the change of protein activity with increasing temperature for all three substrates, within the range allowed by the ITC apparatus (Table 4, Figure 8). As can be seen from Figure 8C, in this temperature range the optimal enzyme efficiency was not achieved for either of the substrates and it probably lies above 75 °C. Temperature had a much stronger effect on the enzyme’s efficiency for (GlcNAc)_2_. Although the enzyme’s turnover number increased with temperature, it was always larger for (GlcNAc)_2_ than for GlcNAc or (GlcNAc)_3_. The shape and size of the substrate-binding pocket also seems to be well suited to accommodate (GlcNAc)_2_ (Figure 5), whereas the smaller compound would not fit in the binding cavity as tightly and its binding specificity is expected to be additionally reduced at elevated temperatures due to diffusion. On the other hand, ligands larger than (GlcNAc)_2_ would protrude from the binding cavity and the hydrogen bond with Gly255* could not form due to the glycosidic bond between the second and third sugar moiety. It is also possible that at higher temperatures the protein would exhibit more flexibility to accommodate larger substrates. This could explain why the reaction with (GlcNAc)_3_ is not observed at temperatures lower than 55 °C.

It is of note that *Pch*-Dac processes (GlcNAc)_2_ with a similar K_M_ as the deacetylase from *P. horikoshii* [6].

In addition, experiments were performed on two monoacetyl derivatives of (GlcNAc)_2_ to determine the activity of *Pch*-Dac toward the acetate groups at the nonreducing and reducing ends of the sugar substrate. Calorimetric ITC measurements to determine the total enthalpy of the conversion of substrate into product, H_app_, were performed with GlcNAc-GlcN and GlcN-GlcNAc tested as substrates. Reaction was observed only in the case of GlcNAc-GlcN, i.e., the enzyme removes the acetate group only at the non-reducing end of the sugar, confirming its assignment to the CE-14 family of enzymes. For this compound, the heat of the injection of the substrate to the protein was greater than in the blank experiment and the heat peak profile was asymmetric with the extended return to the baseline (Figure S5). The specificity of *Pch*-Dac for the acetate group at the non-reducing end of the sugar was confirmed by NMR (see Section 2.6).

### 2.6. Identifying the Reaction Products by NMR Spectroscopy

**Figure 9:**
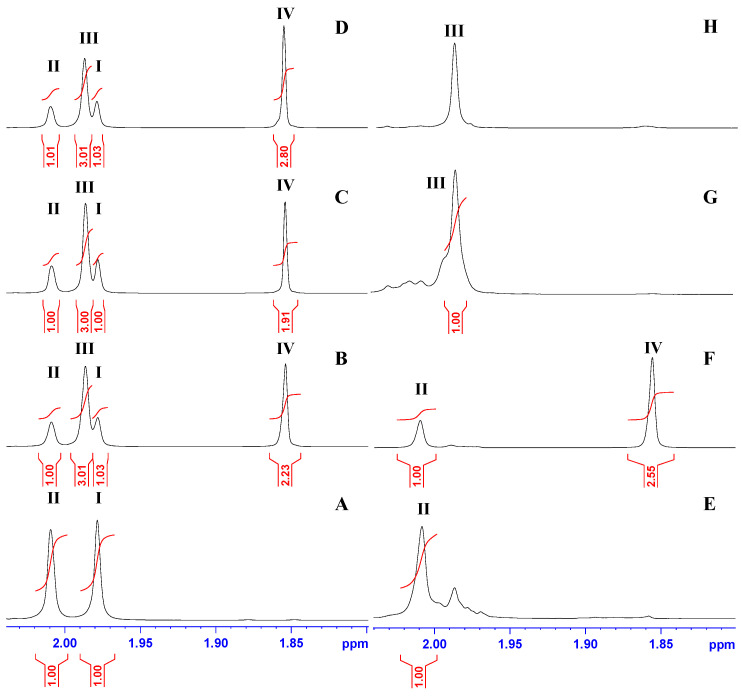
Representative ^1^H NMR spectra of (GlcNAc)_2_ in the methyl region (**A**) and after incubation with *Pch*-Dac for 15 min, 1 h and 3.5 h in HEPES buffer (**B**–**D**). Parts (**E**,**G**) show ^1^H NMR spectra of GlcNAc-GlcN and GlcN-GlcNAc before reaction with the enzyme, respectively. Parts (**F**,**H**) represent ^1^H NMR spectra of GlcNAc-GlcN and GlcN-GlcNAc after incubation with the enzyme for 3.5 h, respectively. Peak (**I**) is assigned to the methyl group of the reducing end of (GlcNAc)_2_, (**II**) the methyl group of the non-reducing end of (GlcNAc)_2_, (**III**) the methyl group of the reducing end of GlcN-GlcNAc, IV: the methyl group of CH_3_COOH.

The analysis of the ^1^H and ^13^C NMR spectra of samples in buffer HEPES/D_2_O upon the reaction of (GlcNAc)_2_ with *Pch*-Dac revealed a mixture of the substrate and products. The complexity of the ^1^H and ^13^C NMR spectra of post-reaction samples meant that reliable analysis of reaction progress on the basis of NMR spectra was possible only up to a narrow range of resonances corresponding to the methyl of *N*-acetyl groups and the resulting CH_3_COOH (Figure 9 and Figure S6). Thus, two proton signals at 2.009 and 1.978 ppm corresponded to the methyl of *N*-acetyl groups of the non-reducing (II) and reducing ends (I) of the substrate, respectively, and two other signals at 1.980 and 1.848 ppm corresponded to the methyl (III) of *N*-acetyl groups of the product and methyl (IV) of CH_3_COOH, respectively (Figure 9). Therefore, these proton resonances were selected as reliable diagnostic signals for a quantitative estimate of the ratio of the substrate and products in the mixture. Moreover, a thorough inspection of the methyl region of ^13^C NMR spectra (Figure S6) i.e., 22.0–23.5 ppm yielded qualitative information on the composition of post-reaction samples that complete the observation provided by the ^1^H NMR spectra.

After 3.5 h of the enzymatic reaction, the ratio of III methyl to I/II methyl groups was approximately 3:1 indicating the predominance of the product 1,4-β-d-glucosaminyl-d-*N*-acetylglucosamine (GlcN-GlcNAc) over the substrate (GlcNAc)_2_ in the mixture (Figure 9B–D). This is in agreement with the published literature where GlcN-GlcNAc was observed as the product [5,11]. Interestingly, the product-to-substrate ratio was similar regardless of the reaction time, which suggested that the reaction reached an equilibrium within 15 min. In order to confirm our observation, we subjected the two monoacetyl derivatives of chitobiose to the enzymatic reaction. The ^1^H spectra of GlcNAc-GlcN and GlcN-GlcNAc after incubation with the enzyme for 3.5 h are presented on Figure 9F and Figure 9H, respectively. The lack of methyl (IV) of CH_3_COOH on Figure 9H clearly showed the stability of *N*-acetyl groups at the reducing end in GlcN-GlcNAc, compared with GlcNAc-GlcN, where signal IV was present, thereby confirming the susceptibility of *N*-acetyl groups of the non-reducing end to the enzymatic reaction.

## 3. Materials and Methods

### 3.1. Gene Cloning, Expression and Protein Purification

*Pyrococcus chitonophagus* DSM 10152 (formerly *Thermococcus chitonophagus)* gene chiton_0574 coding the enzyme diacetylchitobiose deacetylase (*Pch*-Dac) (GenBank code: CUX77353.1) was cloned into T7 expression vector pET151D-TOPO with the N-terminal tag containing the codons for 6xHis and TEV cleavage site. The gene sequence was verified by sequencing (Genomed S.A., Warsaw, Poland). The gene was expressed in BL21-Magic *E. coli* cells in LB medium with 100 mg/mL ampicillin and 25 mg/mL kanamycin. Gene expression was induced with 0.5 mM IPTG and after 4h in 37 °C, the bacterial cells were pelleted and frozen at 80 °C for future use. The cells were resuspended in 50 mL of Buffer C (50 mM TRIS pH = 7.5, 200 mM NaCl). Before cell lysis, the following components were added: 50 mM imidazole, 10% glycerol, 0.5% Triton X100 and 1 mM PMSF. The cells were sonicated on ice for a total time of 4.5 min, with pauses for sample cooling, followed by centrifugation at 16,000 rpm for 30 min. at 4 °C. The supernatant was applied on the His-bind affinity column (Ni resin) and the protein was eluted with 200 mM imidazole in the Buffer C. The eluted protein was dialyzed overnight in Buffer C and in the presence of TEV protease, to remove the imidazole and cut His-tag label. The material was reapplied to the Ni column to remove any protein with the His-tag still attached. *Pch*-Dac was then dialyzed in 25 mM TRIS pH 7.5, 200 mM NaCl and 0.2 mM ZnCl_2_ and concentrated to 4–5 mg/mL using Amicon*^®^* Ultra centrifugal units, ready for crystallization.

### 3.2. Protein Crystallization, Data Collection, Processing, Structure Solution and Refinement

*Pch*-Dac-lig crystallized in space groups P2_1_ in the presence of (GlcNAc)_2_ added in the 5:1 molar excess of the ligand with respect to the protein, and in 0.2 M ammonium chloride, 0.1 M MES pH 6.0, 20% *v/v* PEG 6000, at 18 °C. *Pch*-Dac (unliganded) crystallized in space group P3_2_21 in 2.0 M ammonium sulfate, 0.1 M HEPES, pH 7.5, 2% *v/v* PEG 400, at 35 °C. Crystals for the anomalous dispersion measurements were obtained from purified *Pch*-Dac-anom treated with a 1000:1 molar excess of TPEN, for 1.5 h, followed by a 3-step dialysis (1 h, 4 h and overnight). The protein was then incubated in ZnCl_2_ in 10:1 molar excess of the metal cation. The protein crystallized in space group P2_1_2_1_2_1_ in 2.0 M ammonium sulfate, 0.1 M HEPES, pH 7.5, 2% *v/v* PEG 400, at 35 °C, similarly to the trigonal *Pch*-Dac crystals. The synchrotron X-ray diffraction data were collected at the temperature of 100 K, at beamline P13 operated by EMBL Hamburg at the PETRA III storage ring (DESY, Hamburg, Germany) [12]. Prior to freezing, the crystals were immersed in cryoprotecting solution obtained by mixing the reservoir solution with ethylene glycol, to the final concentration of the latter of 25% *v*/*v*. (GlcNAc)_2_ or Zn^2+^ were added to the cryosolutions, as appropriate for *Pch*-Dac-lig and *Pch*-Dac-anom, to prevent their disassociation from the protein.

*Pch*-Dac and *Pch*-Dac-lig X-ray data were collected at a convenient fixed X-ray energy, whereas the *Pch*-Dac-anom data collection was preceded by an energy scan to determine the X-ray fluorescence peak as the appropriate energy for measuring anomalous dispersion. The X-ray diffraction data were processed using the XDS program [13]. The X-ray data are summarized in Table 5. The structures were solved using Phaser [14] from the CCP4 program suite [15]. The structure of *Pch*-Dac was solved by molecular replacement using the PDB entry 4XM0 as the starting model [7]. The structures of *Pch*-Dac-lig and *Pch*-Dac-anom were solved using the *Pch*-Dac as the starting model. The anomalous density maps for *Pch*-Dac-anom were calculated using as Fourier coefficients the anomalous amplitudes of the reflections, |F^+^-F^−^|, and phases calculated from the refined atomic coordinates and retarded by 90°. The molecular models were built using Coot [16] and refined with Refmac5 [17]. The structures were visualized and analyzed by Chimera, Dali [18] and PISA [19].

### 3.3. Small-Angle X-ray Scattering (SAXS and SEC-SAXS)

For batch SAXS measurements, *Pch*-Dac samples were diluted from the stock solution (4.7 mg/mL) in Buffer A (20 mM TRIS pH 7.4, 200 mM NaCl) to a successive concentration series of 3.48, 2.89, 2.49, 1.92, 1.49, 1.45, 0.91, 0.42, 0.2 mg/mL. For SEC-SAXS, the stock solution was used without dilution. Both protein samples and buffer were 0.22 μm filtered before the SAXS measurements. The concentrations were measured on the NanoDrop Spectrophotometer ND-1000 at A280 nm. Bovine serum albumin (BSA) samples [21] were prepared by directly dissolving the BSA powder (Sigma-Aldrich, Saint Louis, MO, USA, catalogue No. 05470) in different volumes of BSA buffer (25 mM HEPES, 50 mM NaCl, 3%(*v/v*) glycerol pH 7), to the final concentrations of 2.13 and 4.28 mg/mL.

SAXS measurements on the samples were performed at the P12 BioSAXS beamline of PETRA III, DESY (Hamburg, Germany) [22] using both a robotic sample changer and a size exclusion chromatography setup (SEC-SAXS). Prior to the measurements of the deacetylase solutions, BSA samples (2.13 mg/mL for batch mode SAXS and 4.28 mg/mL for SEC-SAXS) were run to optimize the beam conditions. The measurements were conducted in an in-vacuum capillary, with a diameter of 0.9 mm at the X-ray wavelength of *λ* = 0.124 nm and the scattering was recorded on a Pilatus 6 M detector positioned at 3 m from the sample. The covered range of the scattering vector was 0.03 < *s* < 7.0 nm^−1^ with s = 4πsin 2θ/λ, where 2*θ* is the scattering angle. Potential radiation damage effects were reduced through continuous flow of the sample.

In the batch mode, the samples were loaded by the automatic sample changer [23] with a volume of 30 μL and exposed at room temperature to the beam with 0.1 sec per image (40 images in total per sample or buffer). For SEC-SAXS setup [24] the measurements were performed using an Agilent 1260 Infinity II Bio-inert LC system and Superdex 200 Increase 5/150 GL (3 mL) column. The samples were injected at 4 °C with a volume of 30 μL at a flow rate of 0.35 mL/min. and exposed to the beam with 0.25 sec per image (2880 images in total). All exposures of the samples and buffers were checked against the radiation damage, averaged, and subtracted using a SASFLOW pipeline [25,26]. The samples and buffer data frames from SEC-SAXS measurements were analyzed by CHROMIXS [27]. The data was analyzed using the programs from the ATSAS package [28]. The scattering from the high-resolution models was computed using CRYSOL [29] and the volume fractions of hexamers and dodecamers were evaluated by OLIGOMER [30]. The radius of gyration *R_g_* was determined from the scattered intensity I(s) using the Guinier approximation [31].

$$I\left(s\right)\approx I\left(0\right)\exp\left(\frac{-s^{2}R_{g}^{2}}{3}\right),$$

The molecular mass was estimated using the Bayesian inference method [25].

### 3.4. Differential Scanning Calorimetry (DSC)

The thermal stability of *Pch*-Dac was studied by the MICROCAL PEAQ-DSC system (Malvern Instruments Ltd., Malvern, UK). Standard DSC experiment consisted of two measurements, both at the same instrument conditions: (a) five reference scans with buffer-filled cells to establish the instrument thermal history and to achieve a good baseline repeatability; (b) one sample-buffer scan to obtain melting temperature data for analysis.

In order to examine the reversibility of the transitions under study, an extended DSC experiment was also performed in which all scans consisted of heating and cooling steps and both measurements (a and b) were cycled five times.

Before every DSC experiment, the protein sample was dialyzed against the specified buffer, which was later used also in DSC scans, and the protein concentration was measured using NanoDrop. A few preliminary DSC scans were done to establish the best experiment conditions. All the tested conditions were gathered in Table 6.

### 3.5. Microcalorimetric Measurements of Pch-Dac Interactions with Divalent Cations

The isothermal titration calorimetric (ITC) measurements of interactions between *Pch*-Dac and divalent cations were conducted by PEAQ-ITC calorimeter (Malvern). The protein, after TPEN procedure, kept at the concentration of ~110 µM in the sample cell was titrated with 1.2 mM Zn^2+^, Cd^2+^ or Ni^2+^ (in the syringe). The measurements were conducted in 25 mM TRIS buffer pH 7.5 with 200 mM NaCl. The ligand was injected in 19 aliquots of 2 µL. Raw ITC data were analyzed with the *Origin* 7.0 software (Origin-Lab, Northampton, MA, USA) to obtain thermodynamic parameters: stoichiometry (*N*), dissociation constant (*K*_d_) and changes in the enthalpy (Δ*H*) and entropy. ‘One set of binding sites’ model was fitted to the data. Reference power was set to 5. A stirring speed of 750 rpm and spacing of 150 s was used. Measurements were carried out in duplicate.

### 3.6. Microcalorimetric Measurements of Pch-Dac Enzyme kinetics

Kinetic parameters of the deacetylation reaction were measured using ITC in the multiple injection mode (MIM) [32] and MicroCal PEAQ ITC calorimeter (Malvern). The MIM method consists of two separate experiments. First, the enthalpy of total conversion of all the substrate into product (H_app_ i.e., total molar enthalpy of the reaction) was determined by injecting 2 µL of the substrate with the syringe: GlcNAc (kept at 100 mM concentration), (GlcNAc)_2_ (50 mM) or (GlcNAc)_3_ (10 mM) into the reaction cell containing 2 µM enzyme. 3–4 injections were performed separated by 10 min intervals ensuring a total substrate conversion. After peak integration, the values were averaged to obtain H_app_. Next, the differential power change (dQ/dt) arising from the turnover of the substrate into the product was determined in a heat rate shift experiment, in which the substrates in the syringe at 400 mM, 150 mM, or 75 mM concentration (for GlcNAc, (GlcNAc)_2_, or (GlcNAc)_3_ respectively) were injected in nineteen 2 µL aliquots with short 60 s intervals (to minimize substrate depletion) into the cell with the enzyme kept at 50 nM concentration (in case of (GlcNAc)_2_ and (GlcNAc)_3_) or at 190 nM in the reaction with GlcNAc. The kinetic experiments for the (GlcNAc)_2_ substrate were conducted in CHC buffer system: citric acid, HEPES and CHES (2:3:4 molar ratio), pH 4.0 adjusted with NaOH at three different pH conditions (6.5, 7.5 and 8.5) at 75 °C. For the optimal pH (7.5), additional kinetic measurements were carried out for (GlcNAc)_2_ at 25, 35, 45, 55 and 65 °C to trace the change of the enzyme efficiency with the temperature. The above mentioned temperature dependency was studied also for GlcNAc, but in pH 6.5, which appeared to be optimal for this substrate. For (GlcNAc)_3_, kinetic parameters at different temperatures were determined at pH 6.5. The raw rate data were analyzed using the Microcal PEAQ-ITC Analysis Software with the fitting model ‘Enzyme Kinetics-Multiple Injection’. Briefly, they were transformed into reaction rates and substrate concentrations and fitted to the Michaelis-Menten equation. Measurements were taken with stirring at 600 rpm and differential power set to 5 µcal/s. For the hypothetical substrates—GlcNAc-GlcN and GlcN-GlcNAc, due to low availability of the substances, only H_app_ experiments were conducted by injecting 2 µL of 25 mM substrate to 2 µM protein in the cell (at 75 °C and pH 6.5), to compare the heat from the possible reaction with the heat from a blank experiment (i.e., injecting the same amount of the substrate into the buffer).

### 3.7. NMR Spectroscopy

The ^1^H and ^13^C NMR experiments were conducted at 25 °C in HEPES buffer containing 10% D_2_O on a Bruker Avance III (500 MHz) equipped with 5 mm broad-band multinuclear probe (PABBO). The chemical shifts were referenced to internal 4,4-dimethyl-4-silapentane-1-sulfonic acid (DSS). The samples of analyte for the NMR measurements were prepared in at concentration of 50 mM in HEPES buffer (pH 7.5) containing 10% D_2_O (*w/w*). The water signal was suppressed using excitation sculpting with gradients experiment [33]. The ^1^H NMR spectra consisted of 64K sampling points covering a spectral width of 10 ppm. The relaxation delay was set at 2 s, and 100 scans were accumulated for each spectrum. This acquisition was repeated every 6.55 s. The ^13^C NMR spectra were measured using 1D sequence with power gated decoupling using 30° flip angle. The number of sampling points and spectral width was set at 64K and 210 ppm, respectively.

## 4. Conclusions

The extended study of *Pch*-Dac revealed its molecular structure in the crystal and in solution, its thermodynamic stability, enzymatic activity under varying conditions, and substrate specificity. *Pch*-Dac was consistently hexameric in three different crystal forms and in solution, which implied that this was the biologically relevant assembly. The oligomeric structure and individual subunits remained stable up to temperatures slightly exceeding 100 °C. The identity of the bound metal cation was confirmed to be Zn^2+^, although other metal cations were found to also bind with comparable affinities. The enzyme showed a relatively high activity toward (GlcNAc)_2_, the main product of chitin degradation by chitinases, but (GlcNAc) and (GlcNAc)_3_ were also processed, albeit with lower efficiency. Only the acetyl group at the non-reducing end of the sugar was cleaved, with the sugar ring being held in the enzyme’s binding cavity by a network of hydrogen bonds. The activity of *Pch*-Dac showed a rather low dependence on pH in the range 6.5–8.5, but it was highly dependent on temperature, the optimum being above 75 °C, the working limit of the measuring apparatus. The highest recorded efficiency constant, 70 s^−1^·mM^−1^, approaches the values of some common hydrolytic enzymes.

The crystallographic study afforded an analysis of the enzyme in complex with its substrate. This was possible probably due to an excess of the substrate while the enzyme was far from its optimal working conditions (the efficiency of this hyperthermophilic enzyme drops fast with temperature). A comparison of the enzyme-substrate complex with the unliganded enzyme indicated that the Zn^2+^ cation is tetrahedrally coordinated. Three of its ligands are provided by the protein’s conserved His-Asp-His triad and the fourth, in an unliganded structure, is a single water molecule wedged between two catalytic residues, His259* and Asp42. The substrate approaches the Zn^2+^ ion in a monodentate manner, displacing the water molecule with the oxygen atom of its acetyl group. The structure of the protein does not change significantly upon the substrate binding. The *N*-acetyl group is positioned between the two active residues, approximately 3.5 Å from each. There is no evidence in the electron density of a water molecule that could act as the hydrolytic water and there is no place where a water molecule could be fitted in the immediate vicinity of the active site. This does not mean that water could not penetrate the complex, but this crystal structure does not show how this could happen. One needs to allow for the active enzyme showing dynamics not seen in the crystal structure and hydrolysis as the first step cannot be ruled out, but this structure is consistent with the reaction mechanism that involves an anhydride intermediate.

## Figures and Tables

**Table 1:** Thermodynamic parameters of binding different divalent cations to *Pch*-Dac.

	Zn^2+^	Cd^2+^	Ni^2+^
Stoichiometry	0.86 ± 0.02	0.87 ± 0.02	0.62 ± 0.01
K_d_ [µM]	9.5 ± 1.3	11.0 ± 1.3	12.7 ± 1.0
ΔH [cal/mol]	−8666 ± 314	−15,040 ± 498	−12,400 ± 332
ΔS [cal/mol/deg]	−6.1	−27.8	−19.2

**Table 2:** Kinetic parameters of *Pch*-Dac activity toward GlcNAc, (GlcNAc)_2_ and (GlcNAc)_3_, at 75 °C and pH 6.5.

Substrate	K_m_ [mM]	k_cat_ [s^−1^]	k_cat_/K_m_
GlcNAc	22.4 ± 1.5	226 ± 6	10 ± 1
(GlcNAc)_2_	2.6 ± 0.2	178 ± 2	68 ± 6
(GlcNAc)_3_	11.2 ± 1.1	71 ± 4	6 ± 1

**Table 3:** Kinetic parameters of (GlcNAc)_2_ deacetylation at different pH conditions, at 75 °C.

pH	K_M_ [mM]	k_cat_ [s^−1^]	k_cat_/K_M_
6.5	2.6 ± 0.2	178 ± 2	68 ± 6
7.5	3.7 ± 0.1	263 ± 1	71 ± 2
8.5	4.8 ± 0.4	169 ± 4	35 ± 4

**Table 4:** Kinetic parameters for GlcNAc, (GlcNAc)_2_ and (GlcNAc)_3_ deacetylation at different temperatures. The ‘-‘ signs indicate no detectable heat effect.

Substrate	Temp. (°C)	25	35	45	55	65	75
GlcNAc	K_M_ [mM]	-	9.2 ± 0.2	19.5 ± 1.1	14.8 ± 0.4	13.3 ± 0.4	22.4 ± 1.5
	k_cat_ [s^−1^]	-	32.6 ± 0.2	75.1 ± 1.6	117 ± 1	102 ± 1	226 ± 6
	k_cat_/K_M_ [s^−1^·mM^−1^]	-	3.5 ± 0.1	3.8 ± 0.3	7.9 ± 0.3	7.7 ± 0.3	10.2 ± 0.9
(GlcNAc)_2_	K_M_	3.8 ± 0.2	6.3 ± 0.2	4.3 ± 0.1	4.5 ± 0.2	5.1 ± 0.2	3.7 ± 0.1
	k_cat_	23.4 ± 0.4	53.1 ± 0.5	84.3 ± 0.3	153 ± 2	222 ± 3	263 ± 2
	k_cat_/K_M_	6.2 ± 0.4	8.4 ± 0.3	19.6 ± 0.5	34 ± 2	44 ± 2	71 ± 2
(GlcNAc)_3_	K_M_	-	-	-	14.2 ± 1.3	11.1 ± 1.1	11.2 ± 1.1
	k_cat_	-	-	-	31.4 ± 1.8	48.1 ± 2.8	70.6 ± 4.2
	k_cat_/K_M_	-	-	-	2.2 ± 0.3	4.3 ± 0.7	6.3 ± 1.0

**Table 5:** Summary of the X-ray data and the final models.

Protein	*Pch*-Dac-lig	*Pch*-Dac	*Pch*-Dac-anom ^‡^
PDB code	8BGO	8BGN	8BGP
Space group	P2_1_	P3_2_21	P2_1_2_1_2_1_
Unit cell parameters:			
*a* (Å)	79.2	150.6	78.6
*b* (Å)	151.7	150.6	158.7
*c* (Å)	167.1	72.2	158.6
α (°)	90	90	90
*β* (°)	93.3	90	90
γ (°)	90	120	90
Wavelength (Å)	0.9797	0.9797	1.2815
Resolution (Å)	3.08	2.76	2.51
R_merge_ *^,#^	0.077 (0.657)	0.114 (0.966)	0.199 (1.090)
R_pim_ ^†^			0.057 (0.303)
Completeness (%)	99.3	99.08	94.2
Observed reflections	488,298	470,509	565,577
Unique reflections	6943	24,234	43,577
<I/σ(I)>	14.6 (2.0)	18.4 (2.4)	11.1 (3.03)
Protein subunits/asymmetric unit	12	3	6
R ^§^/R_free_^$^	0.1687/0.2147	0.1763/0.2271	0.2183/0.2614

^‡^ Diffraction data were processed and statistics calculated by STARANISO [20]. * Values in brackets are for the highest resolution shell. ^#^ R_merge_ = Σ_hkl_Σ_i_|I_i_(hkl) − <I(hkl)>|/Σ_hkl_Σ_i_I_i_(hkl), where I_i_(hkl) is the integrated intensity of a given reflection and <I(hkl)> is the mean intensity of multiple corresponding symmetry-related reflections. ^†^ R_pim_ = Σ_hkl_(1/n − 1)^1/2^Σ_i_|I_i_(hkl) − <I(hkl)>|/Σ_hkl_Σ_i_I_i_(hkl). ^§^ R = Σ_hkl_||F_obs_| − |F_calc_||/Σ_hkl_ |F_obs_|, where F_obs_ and F_calc_ are the observed and calculated structure factors, respectively. ^$^ R_free_ is R calculated using a randomly chosen subset of reflections excluded from the refinement.

**Table 6:** DSC experimental conditions for the thermal unfolding of *Pch*-Dac.

Sample Concentration	0.70 mg/mL (22 µM), 1.24 mg/mL(40 µM), 1.39 mg/mL (45 µM), 1.79 mg/mL (58 µM)
Sample Solution Components	Buffer A: 20 mM TRIS pH 7.4, 200 mM NaCl Buffer B: 25 mM HEPES pH 7.5, 200 mM NaCl, 10 µM ZnCl_2_
Scan Rates	45 °C/h, 60 °C/h, 90 °C/h, 240 °C/h.
Feedback Modes	high, medium

## Data Availability

The coordinates and structure factors have been deposited in the Protein Data Bank under Accession Codes 8BGN, 8BGO and 8BGP.

## Supplementary Materials

The following supporting information can be downloaded at: https://www.mdpi.com/article/10.3390/ijms232415736/s1.

## Abbreviations

*Pch*-Dac	*N*:*N*-diacetylchitobiose deacetylase from *Pyrococcus chitonophagus*
*Pch*-Dac-lig	Crystal structure of *Pch*-Dac with bound substrate
*Pch*-Dac-anom	Crystal structure of *Pch*-Dac used for identifying the prosthetic group
GlcNAc	*N*-acetyl-d-glucosamine
(GlcNAc)_2_	1,4-β-d-*N*-acetyl glucosaminyl-d-*N*-acetyl glucosamine
GlcN-GlcNAc	1,4-β-d-glucosaminyl-d-*N*-acetylglucosamine
GlcNAc-GlcN	1,4-β-d-acetyl glucosaminyl-d-*N*-glucosamine
r.m.s.d.	root-mean-square deviation
DSC	Differential Scanning Calorimetry
ITC	Isothermal Titration Calorimetry
TPEN	*N*,*N*,*N*′,*N*′-tetrakis(2-pyridinylmethyl)-1,2-ethanediamine; ion chelator
DSS	4,4-dimethyl-4-silapentane-1-sulfonic acid; standard used in NMR
