# Supplementary material for: Structural, Thermodynamic and Enzymatic Characterization of N,N-Diacetylchitobiose Deacetylase from Pyrococcus chitonophagus
Source: Int J Mol Sci. 2022 Dec 12;23(24):15736. doi: 10.3390/ijms232415736 (PMC9779004; doi:10.3390/ijms232415736)
Supplement: Supplementary file 1 [file ijms-23-15736-s001.zip › ijms-2028736-supplementary.pdf]

Supplementary Material

to

Structural, Thermodynamic and Enzymatic Characterization of *N,N*-Diacetylchitobiose Deacetylase from *Pyrococcus chitonophagus*

Katarzyna Biniek-Antosiak [1], Magdalena Bejger [1], Joanna Śliwiak [1], Daniel Baranowski [1], Ahmed S. A. Mohammed [2], Dmitri I. Svergun [2] and Wojciech Rypniewski [1,*]

[1] Institute of Bioorganic Chemistry, Polish Academy of Sciences, Noskowskiego 12-14, 61-704 Poznań, Poland

[2] European Molecular Biology Laboratory (EMBL), Hamburg Unit, DESY, Notkestrasse 85, D-22607 Hamburg, Germany

* Correspondence: wojtekr@ibch.poznan.pl

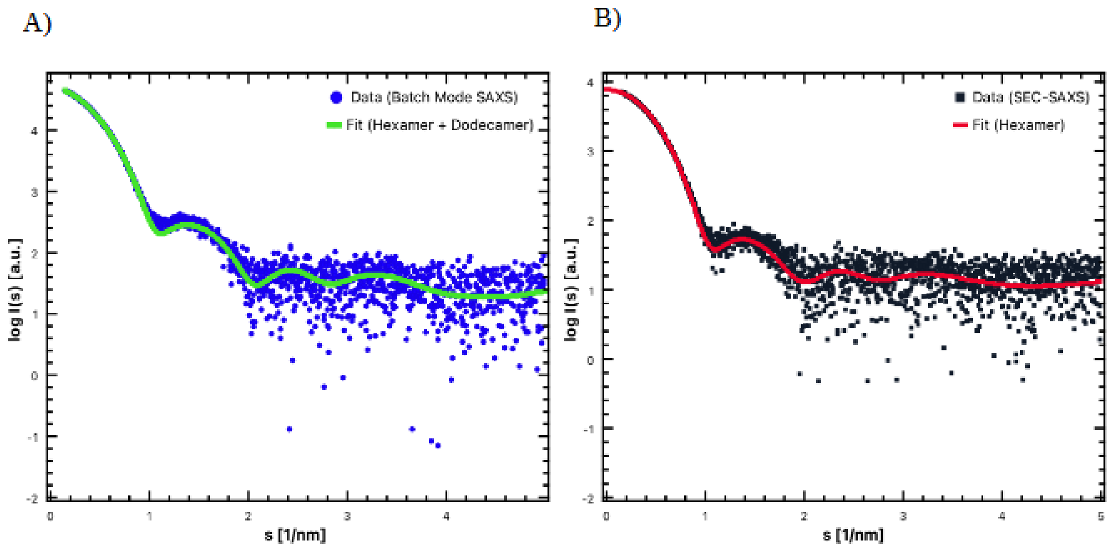

Figure S1. (A) Scattering data from *Pch*-Dac at 4.7 mg/ml collected in batch mode (*blue*) and the scattering from a mixture of hexamers and dodecameric assemblies computed by OLIGOMER (*green*); (B) SEC-SAXS data from the main elution peak of deacetylase (*black*) and the intensity from the hexamer model computed by CRYSOL (*red*).

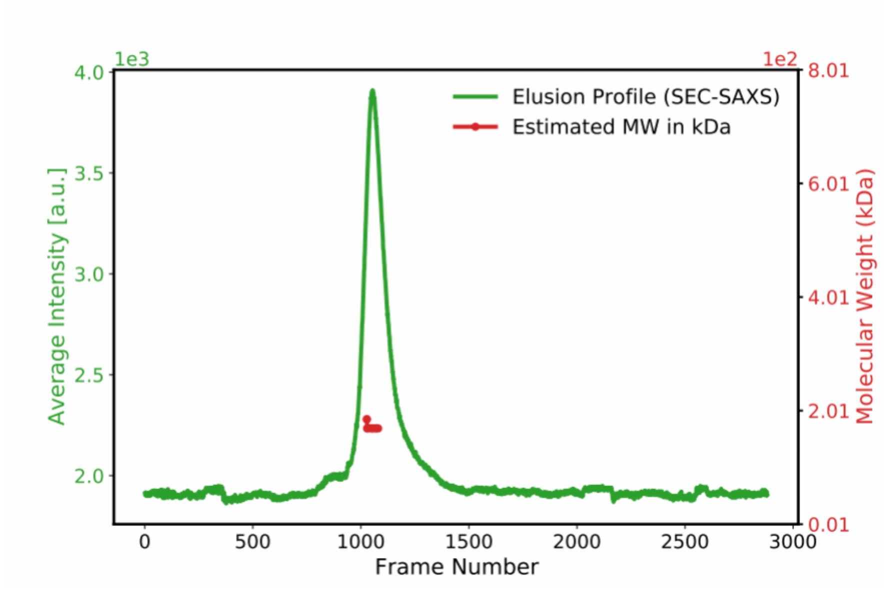

Figure S2. SEC-SAXS elution profile of *Pch*-Dac computed by CHROMICS and displaying one predominant elution peak. The molecular weight across the peak is displayed in red.

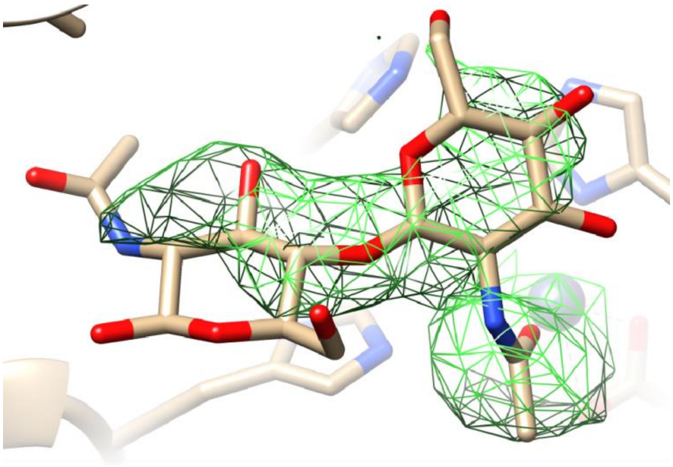

Figure S3. 'Omit map' of $(GlcNAc)_2$ bound to a *Pch*-Dac-lig subunit. It is the 'difference electron density' map calculated after removing the ligand from the atomic model and three cycles of automatic model refinement. The map is contoured at the 3σ level.

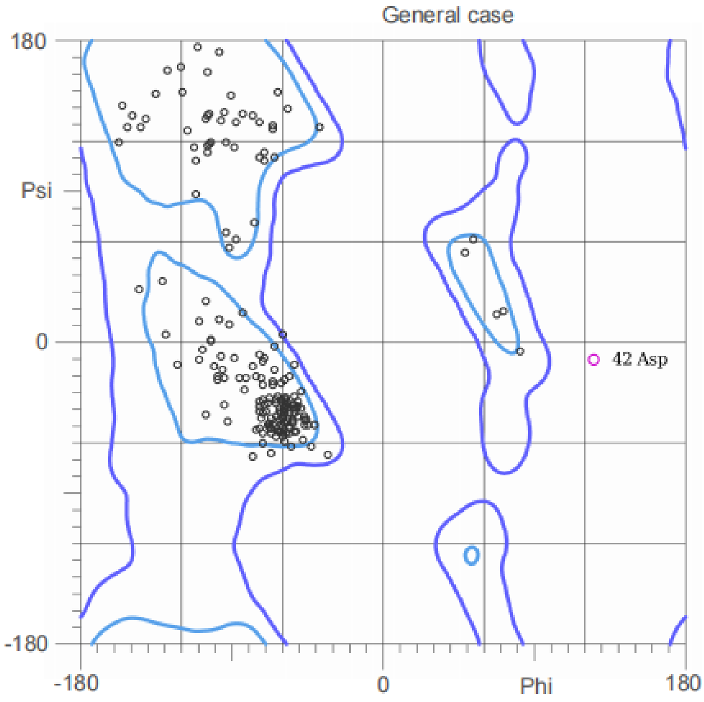

Figure S4. Ramachandran plot of a *Pch*-Dac subunit. The only outlier is Asp42.

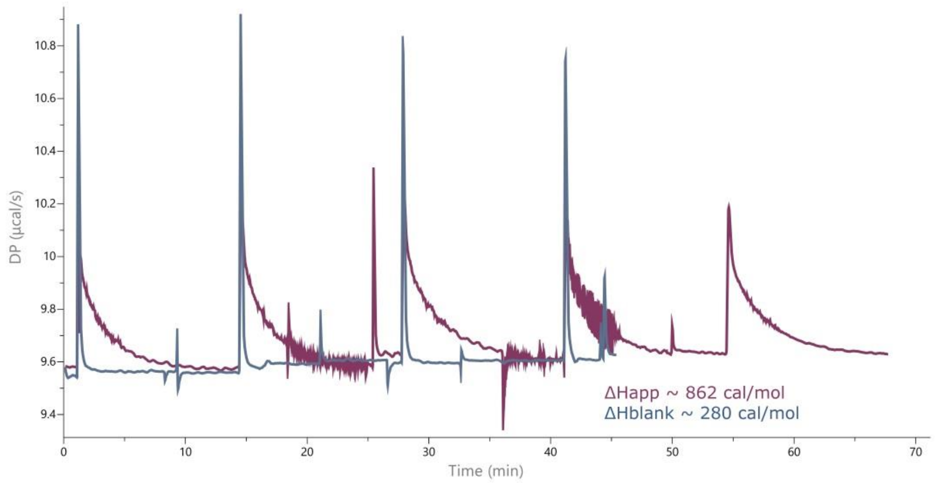

(a)

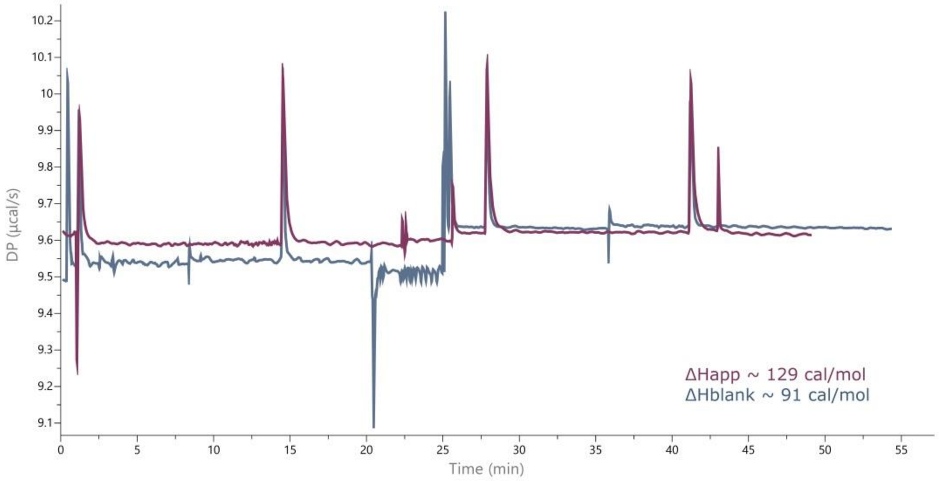

(b)

Figure S5. Results of ΔH_{app} experiments done for *Pch*-Dac with (a) glucosaminyl acetylglucosamine (GlcNAc-GlcN) and (b) acetylglucosaminyl glucosamine GlcN-GlcNAc. Violet line indicates injections of 2 μL of 25 mM substrate to 2 μM protein in the cell (at 75°C and pH 6.5), blue line – injections of 2 μL of 25 mM substrate to the reaction buffer in the cell.

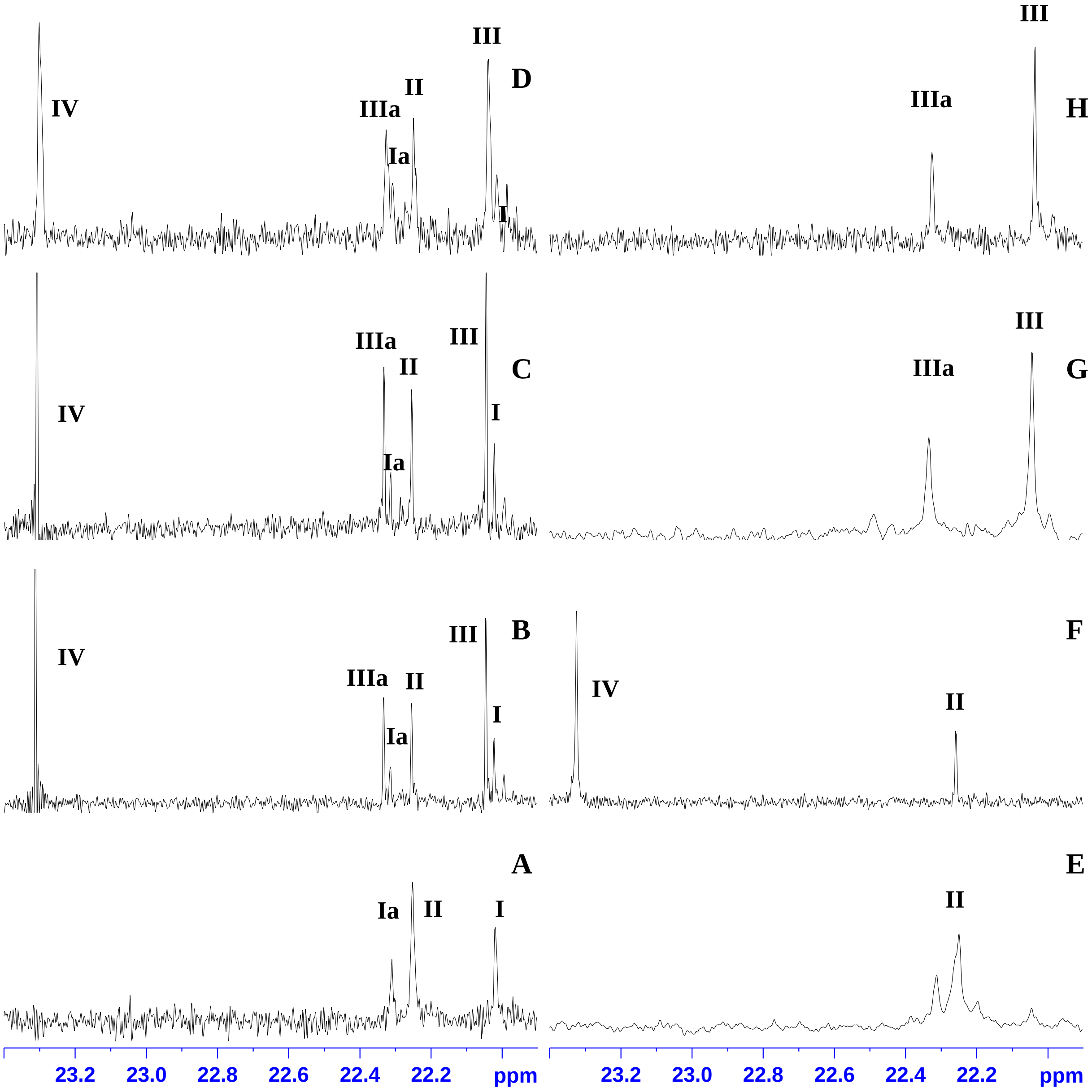

Figure S6. Representative ^{13}C NMR spectra of $(GlcNAc)_2$ in the methyl region (A) and after incubation with *Pch*-Dac for 15 min, 1 h and 3.5 h in HEPES buffer (B, C and D). Parts E and G represent ^{13}C NMR spectra of GlcNAc-GlcN and GlcN-GlcNAc before the reaction with the enzyme, respectively. Parts F and H represent ^{13}C NMR spectra of GlcNAc-GlcN and GlcN-GlcNAc after incubation with the enzyme for 3.5h, respectively. Peak I is assigned to the methyl group of the reducing end of $(GlcNAc)_2$, Ia: the methyl group of the reducing end of a second anomer of GlcN-GlcNAc, II: the methyl group of the non-reducing end of $(GlcNAc)_2$, III: the methyl group of the reducing end of GlcN-GlcNAc, IIIa: the methyl group of the reducing end of a second anomer of GlcN-GlcNAc IV: the methyl group of CH_3COOH

Table S1. Calculated parameters of *Pch*-Dac solutions in the batch mode of the successive dilution series and of the SEC-SAXS data: radius of gyration (R_g), molecular weight (MW), goodness-of-fit (χ^2), and computed volume fractions of hexamer and 'dodecamer' by OLIGOMER [1].

C [mg/ml]	R_g [nm]	*MW* [kDa]	χ^2	Volume fraction (Hexamer)	Volume fraction ('Dodecamer')
4.7	3.85 ± 0.01	185	1.49	0.87 ± 0.002	0.126 ± 0.001
3.48	3.90 ± 0.01	177	1.46	0.85 ± 0.001	0.142 ± 0.001
2.89	3.89 ± 0.01	185	1.63	0.86 ± 0.002	0.138 ± 0.001
2.49	3.89 ± 0.01	185	1.43	0.86 ± 0.002	0.140 ± 0.001
1.92	3.88 ± 0.01	185	1.08	0.86 ± 0.003	0.137 ± 0.002
1.49	3.93 ± 0.01	185	1.48	0.84 ± 0.003	0.157 ± 0.002
0.91	3.94 ± 0.01	185	1.10	0.84 ± 0.006	0.158 ± 0.005
0.42	3.92 ± 0.01	185	1.10	0.84 ± 0.011	0.151 ± 0.008
0.2	3.87 ± 0.01	170	0.97	0.86 ± 0.008	0.131 ± 0.006
SEC-SAXS	3.71 ± 0.01	177	1.2	1	0

Reference

1. Konarev, P.V.; Volkov, V.V.; Sokolova, A.V.; Koch, M.H.J.; Svergun, D.I. PRIMUS: a Windows PC-based system for small-angle scattering data analysis. *J. Appl. Cryst.* **2003**, *36*, 1277-1282.
